# Clinical and economic ramifications of switching antipsychotics in the treatment of schizophrenia

**DOI:** 10.1186/1471-244X-9-54

**Published:** 2009-09-02

**Authors:** Douglas E Faries, Haya Ascher-Svanum, Allen W Nyhuis, Bruce J Kinon

**Affiliations:** 1 US Statistics, Lilly USA, LLC, Indianapolis, IN, USA; 2 US Outcomes Research, Eli Lilly and Company, Indianapolis, IN, USA; 3 Psychosis Medical, Eli Lilly and Company, Indianapolis, IN, USA

## Abstract

**Background:**

Switching between antipsychotic medications is common in the treatment of schizophrenia. However, data on clinical and economic outcomes from antipsychotic switching, in particular acute care service use, is fairly limited. The goal of this research was to assess the clinical and economic ramifications of switching antipsychotics during outpatient management of schizophrenia.

**Methods:**

Data from a 1-year randomized, open-label cost-effectiveness study involving typical and atypical antipsychotics were assessed. The study protocol permitted switching of antipsychotics when clinically warranted. The risk of crisis-related events, use of acute-care services, and the time to the initial use of such services were determined in outpatients who switched antipsychotics compared with those who continued with their initial medications. Health care resource utilization data were abstracted from medical records and other sources (e.g., patient self-report), and direct costs were estimated using previously published benchmarks.

**Results:**

Almost one-third of patients (29.3%) underwent a switch from their initial antipsychotic agent, with an average duration of 100 days before such treatment alterations. Compared with their counterparts who remained on their initial therapies, individuals who switched antipsychotics experienced a significantly higher risk of acute-care services, including hospitalization (p = .013) and crisis services (p = .011). Patients undergoing medication switches also used acute-care services significantly sooner (p = .004) and accrued an additional $3,000 (a 25% increase) in annual total health care costs per patient, most of which was due to acute-care expenditures.

**Conclusion:**

Switching antipsychotic medications was found to be associated with considerably poorer clinical and economic outcomes, as reflected by, more frequent and more rapid use of acute-care services compared with persons remaining on their initial treatments.

**Trial Registration:**

Trial ID 2325 in LillyTrials.com (also accessible via ClinicalStudyResults.org).

## Background

Antipsychotic medications are mainstays in the management of schizophrenia, yet many patients experience suboptimal improvement (or even worsening) in the core symptoms of their disease or intolerance to their initially prescribed treatments [1-3]. Under such conditions, a clinically indicated change (i.e., switch) in antipsychotics represents a viable treatment option, and such alterations are not uncommon among patients with schizophrenia [3-11]. Clinically warranted switches often confer tangible benefits by enhancing treatment effectiveness and tolerability, and overall treatment acceptance by patients [4,6,11]. Switching antipsychotic medications in an outpatient setting often is a composite decision where 1 or more of the key participants - clinician, patient, or family - decide that the efficacy or tolerability limitations of the current antipsychotic justify the attempt to change medications [9,12].

Data on the clinical and economic ramifications of antipsychotic switching, particularly the use of acute-care services and the time to the first use of such services, are limited. Such information is especially relevant for policy makers and other mental health decision makers. To extend available research, we performed a *post-hoc *analysis using data from a 1-year open label naturalistic cost-effectiveness study of antipsychotics in the treatment of patients with schizophrenia-related disorders, in which switching of antipsychotics was allowed if clinically warranted. As reported in the original study publication [13], approximately one-third of the patients switched antipsychotic medications during the study and such patients had higher total 1-year health care costs than patients who did not switch medications. The objectives of the current analysis were to extend prior findings and compare persons who undergo medication switching with those who do not in terms of rate of acute-care services, the time to the first use of such services, the direct total annual health care cost and the treatment component costs. In addition, we assessed the baseline characteristics of persons who switch medications and their treatment patterns before and following the medication switch.

## Methods

### Study design

This *post-hoc *analysis used data of a 1-year open label randomized cost-effectiveness study of atypical and conventional antipsychotics [13]. Details about the design of the parent study have been published and are available elsewhere [13]. In brief, this study was conducted from May 1998 through September 2001 at 21 sites in 15 states in the United States. Protocol and consent documents were approved by a central institutional review board (IRB) or by local IRBs, and signed consent forms were obtained from patients prior to participation. Patients of age 18 or greater with a DSM-IV diagnosis of schizophrenia, schizoaffective, or schizophreniform disorder [14] and a score of at least 18 on the Brief Psychiatric Rating Scale (BPRS) were eligible [15]. No patient was excluded because of substance abuse disorder or other comorbidities.

At study enrollment, patients were randomly allocated to one of three first-line treatment cohorts: one of two atypical antipsychotics (n = 450) or a conventional antipsychotic of physician's choosing (n = 214). Barring clinically significant adverse events, patients remained on their initial medications for at least 8 weeks, after which they could change medications if a switch was clinically warranted. Medications could also be discontinued, or their doses altered, at each physician's discretion.

### Measures

The primary measures of the current analysis included 3 types of acute-care services, defined as hospitalizations, partial hospitalizations, and visits to emergency departments (EDs). For each service type, we assessed the proportion of patients receiving the service, the rate of use and rate of admissions, the time to first use of the service, and the annual health care cost, in terms of total health care cost and cost components (acute services, medication, other). Data on each patient's use of such services were systematically abstracted from medical records, patient self-reports, and the study sites' administrative databases using a utilization form developed for the study. Cost estimates for each unit of resource use have been previously described in detail [13]. In brief, Medicare public data were used as cost benchmarks for units of specific services and applied to the collected resource use data to obtain costs of care for each patient. Medication costs were based on 2001 average wholesale prices discounted 15% to reflect real-world costs [13]. To standardize costs to a similar period of time for each patient, costs were prorated to a yearly basis by computing the average cost per day for each patient and multiplying this value by 365.

### Statistical analysis

Patients were defined as having switched medications if they discontinued the medication to which they were initially randomized at any time during the study and initiated therapy with a different antipsychotic within 14 days of discontinuation of the initial therapy. Data on the occurrence of acute-care service use, the time to the use of such services, and total-care and acute-care service use costs over 1 year were compared between patients who switched antipsychotics and those who continued with their initial medications.

Patients who discontinued the study prematurely without switching antipsychotics were classified as non-switchers. Because one cannot conclusively determine whether individuals who discontinued the study prematurely also switched medications after leaving the study, a sensitivity analysis was performed in which only patients who completed the study were included. Furthermore, because the study's outcome measures involved the occurrence of acute-care service use, we included only patients who were not using such services at baseline (outpatients). Although patients were randomized to treatments at baseline, analyses between switchers and continuers involved comparisons of groups not formed by randomization. Consequently, analyses were adjusted by propensity score stratification [16,17] based on study site, age, gender, race, baseline BPRS total score, initial therapy, health insurance status, substance abuse diagnosis, duration of any past hospitalizations, and illness duration. These variables were chosen *a priori *based on prior analyses of this study [13].

Acute-care service use was compared using nonparametric bootstrap methods stratified by propensity score quintile. Separate analyses were conducted to determine the number (%) of patients undergoing hospitalization, partial hospitalization/day treatment, crisis services, and any acute-care services; as well as the total duration (in days) of each of these services and the rate of admissions to the facilities providing them. Total and acute care-related component costs were assessed using the same methodology. A nonparametric approach was selected because many of these outcome measures were expected to have highly skewed distributions. Time to acute care-related events was assessed using a Cox proportional hazards model.

Analyses evaluating the impact of excluding patients who were randomized to the medication that they had been taking immediately prior to the trial was performed as an additional sensitivity analysis. Such patients have been previously found to have different outcomes compared to patients randomized to a therapy they were not taking prior to the study [12]. All tests were two-tailed with a level of significance of α = 0.05.

## Results

**Figure 1:**
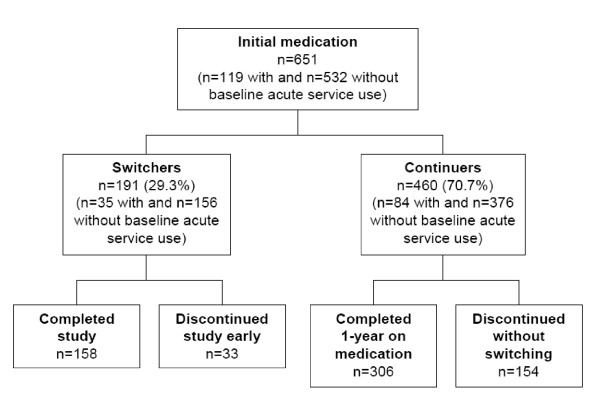
**Patient flowchart**.

Among 664 patients enrolled, 13 (1.9%) did not start the medications to which they were initially randomized and hence were excluded from the present analysis. Of 651 patients with evaluable data, 191 (29.3%) switched antipsychotics, while the remaining 460 (70.7%) continued with their initial medications (Figure 1). A total of 155 individuals who discontinued the study early (prior to completing 1-year) without switching to a different medication were included in the continuer group. When excluding patients with acute-care service use at baseline, 156 patients switched antipsychotics, and 376 continued with their original medications. On average, the duration of treatment with antipsychotic medications before switching was 100 days, while the average duration of treatment after switching was 265 days. Of the switchers, 70 switched from conventional antipsychotics to olanzapine, 41 from risperidone to olanzapine, 15 from olanzapine to conventional, 11 from olanzapine to risperidone, with the rest being other combinations. Following the medication switch, 129 patients completed the study on their new medication and 12 required an additional medication switch during the study. Unfortunately, reasons for medication switching were not obtained for 28.2% of switchers, with the rest evenly divided between patient request (26.3%), lack of efficacy (23.1%), and adverse events (22.4%).

Clinical and demographic characteristics at baseline were fairly similar between switchers and continuers in the analysis sample (Table 1), although a significantly lower proportion of switchers (vs. continuers) were male and the switchers had a lower mean PANSS total score. Switchers also tended to be prescribed lower mean dosages than continuers. For instance, among patients randomized to risperidone, the mean starting doses (2.5 mg/day for switchers vs. 3.0 mg/day for continuers), the modal (4.2 vs. 4.9), and maximum (5.3 vs. 6.3) doses were numerically lower for switchers compared to continuers. For patients randomized to olanzapine, the mean starting doses were similar (8.0 mg/day for switchers vs. 8.2 mg/day for continuers), but the modal (10.0 vs. 13.0) and maximum (13.3 vs. 17.8) doses were lower for switchers.

**Table 1:** Patient baseline characteristics for switchers and continuers of the initial antipsychotic - analysis sample

***Characteristic***	***Patients continuing with their initial antipsychotics (N = 376)***	***Patients switching antipsychotics (N = 156)***	**p-value**
Male, %	66.5	51.9	.002
Age, mean (SD), y	42.6 (12.0)	42.8 (12.8)	.877
Age of onset, mean (SD), y	22.8 (10.3)	22.6 (9.2)	.802
Unemployed	79.0	78.9	.971
Race, %			
Caucasian	54.8	53.9	.843
African descent	33.0	34.0	.824
Other	12.2	12.2	.986
Diagnosis, %			
Schizophrenia	65.7	61.5	.362
Schizoaffective	25.0	28.2	.443
Schizophreniform	9.3	10.3	.735
Comorbidities, %			
MDD	13.3	14.1	.805
Anxiety	6.1	3.2	.171
Substance abuse	32.2	25.8	.146

	***Patients continuing with their initial antipsychotics (N = 376)***	***Patients switching antipsychotics (N = 156)***	**p-value**

Baseline PANSS total, mean (SD)	88.9 (20.6)	84.5 (19.3)	.023
Hospitalization in prior year	30.2	24.5	.191

Abbreviations: MDD, major depressive disorder; PANSS, positive and negative syndrome scale.

P-values based on chi-square and t-tests.

Individuals who switched antipsychotics were significantly more likely to use acute-care services compared with their counterparts continuing with their initial medications. For example, as shown in Table 2, the proportion of patients using any acute-care service and the rate of admission to facilities providing such services were higher among switchers compared with continuers (p < .001). Differences in acute-care service use were driven primarily by differences in hospitalizations and crisis service use as opposed to partial hospitalizations. The proportion of patients hospitalized and the rate of hospitalizations were statistically significantly higher in those who switched. Similar differences were found for crisis service use. There were no statistically significant differences in partial hospitalizations between the two groups.

**Table 2:** Annual acute-care service use^a ^for switchers and continuers on the initial antipsychotic

	***Patients continuing with their initial antipsychotics (N = 376)***	***Patients switching antipsychotics (N = 156)***	**p-value**
Total Days in Study	110,482	52,288	
Mean Days in Study per patient, days	293.8	335.2	
Hospitalization			
% of patients	16.0	26.3	<.001
Duration, days	1215	996	
Rate^b^	1.10%	1.90%	.014
Number of hospital admissions	115	78	
Rate^c^	0.11%	0.15%	.013
Partial hospitalization/day treatment centers			
% of patients	10.6	18.0	.054
Duration, days	3,657	1,561	
Rate^b^	3.35%	3.04%	.491

	***Patients continuing with their initial antipsychotics (N = 376)***	***Patients switching antipsychotics (N = 156)***	**p-value**

Number of admissions to partial hospitals/day treatment centers	65	45	
Rate^c^	0.06%	0.09%	.306
Crisis service			
% of patients	12.0	18.0	<.001
Number of admissions	78	97	
Rate^b^	0.07%	0.20%	.011
Any acute-care service			
% of patients	28.5	42.3	<.001
Number of admissions	258	220	
Rate^c^	0.24%	0.44%	<.001

p values based on propensity score adjusted bootstrap resampling.

^a^Excluding patients with any crisis event at baseline.

^b^Rate = number of days with each service divided by the total number of days in the study.

^c^Rate = number of admissions divided by the total number of days of treatment

Note: the denominator for the rate of partial hospitalization/day treatment days excludes hospitalization days. The denominator for the rate of crisis days excludes hospitalization and partial hospitalization/day treatment days. The denominator for admissions also excludes non-admission hospitalizations and partial hospital/day treatment days.

**Figure 2:**
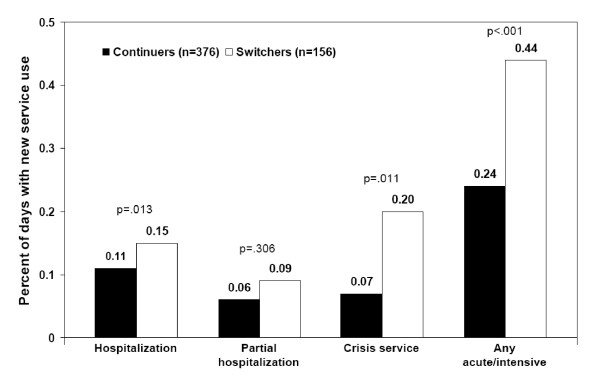
Risk of new acute-care service use (admissions).* *The percentage of days with new service use is calculated by the number of days with a new service (hospital admission, new partial hospitalization treatment, or ER admission) divided by the number of eligible days on treatment. Eligible days on treatment exclude days in which the previous day was spent hospitalized or in partial hospitalization.

**Figure 3:**
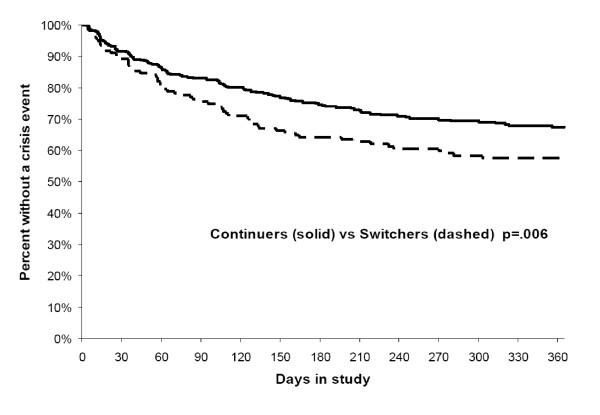
**Time to first acute-care service event**.

As shown in Figure 2, the risk of new acute-care service use (rate of admissions) was significantly higher among individuals switching antipsychotics (vs continuers) for any acute-care service (p < .001), hospitalization (p = .013), and crisis services (p = .011) but not partial hospitalization. Not only did switchers have higher risks of using new acute-care services; they also used such services significantly earlier than continuers (p = .006; Figure 3).

**Figure 4:**
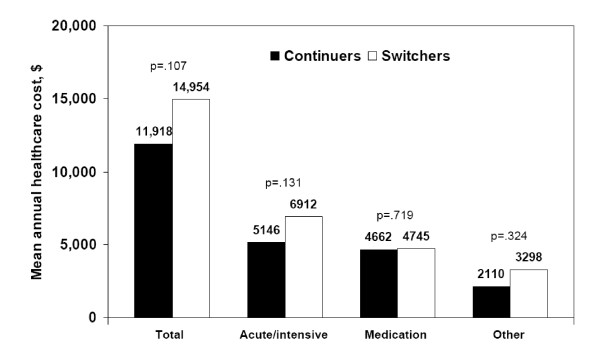
**Average 1-year total health care costs and cost components for switchers and continuers**.

On average, the total annual health care cost of treatment per patient was $14,954 among patients who switched antipsychotics compared with $11,918 for those who continued with their initial treatment (p = .107), translating to an excess of just over $3,000 (or 25%, Figure 4). More than half of this cost difference was attributed to higher expenses for acute- or intensive-care services.

Results of the sensitivity analysis which included only patients who completed the 1-year study were consistent with the above findings. In addition, a total of 68 patients in the analysis dataset had been randomized to the medication they had been taking immediately prior to the trial. The majority of these patients were previously treated with (and were randomized to) conventional antipsychotics (N = 43), with smaller numbers for olanzapine (n = 14) and risperidone (n = 11). The sensitivity analysis excluding such patients did not change the direction nor the statistical significance of any of the findings. For instance, total 1-year costs for switchers and non-switchers after excluding these patients were (N = $12,157 vs. $14,729 for non-switchers vs. switchers). Patients randomized to the same medication that was being taken prior to the trial were found to have a numerically lower, but not significantly different, risk of switching/discontinuation of the randomized medication (HR = 0.89, p = .506).

## Discussion

In this naturalistic 1-year study, switching antipsychotic medication regimens was common (occurring in approximately one-third of patients) and was consistent with previous research in which switching rates have ranged from 25% to 50% [8,11,18]. Findings from our post-hoc analyses show that individuals who switched antipsychotics were significantly more likely to use a range of acute-care services, and did so significantly earlier, compared with those remaining on their initial regimens. These disparities had economic consequences, including about a $3,000 higher annual total health care cost for switchers (vs. continuers). In addition, previous research from this study [19] suggests that the increased use of acute-care services occurred around the time of antipsychotic medication switching [20].

Taken together, findings from the present study and others suggest that switching antipsychotic medications represents a proxy for treatment failure [2,8,9,12]. Although switching often constitutes a sound therapeutic alternative to enhance patient-related outcomes in the face of suboptimal improvement in core symptoms and/or inadequate medication tolerability [4-11], such changes also have heretofore underappreciated clinical and economic ramifications. Given a potentially higher risk of crisis events and attendant acute-care service use around the time of antipsychotic medication switching [20], it appears desirable to avoid switching by tailoring treatment to the individual needs of each patient and closely monitoring the effectiveness, safety, tolerability, and overall patient acceptance of therapy [12,21]. Further research is warranted to identify predictors--either at baseline or early in treatment--of switching antipsychotic medications and to evaluate the effects of switching on other potential patient-related outcomes, including long-term symptom control, overall health status, and quality of life. Although medication switching appears to be a proxy for treatment failure, one may also view switching as a marker of 'difficult to treat' patients. Thus, further research is needed to better identify patients for whom switching will and will not bring beneficial clinical and economic outcomes.

Strengths of the present study include its liberal eligibility criteria and naturalistic treatment setting, which should render the findings generalizable to wide populations of patients with schizophrenia seen in usual outpatient settings [22]. Potential limitations of the study include its *post-hoc *design, which is by nature hypothesis generating rather than hypothesis testing. Our analysis was not adjusted for multiple comparisons, which may increase the likelihood of observing spurious statistically significant differences between switchers and continuers (i.e., false positives).

Furthermore, statistical comparisons between switchers and continuers involved groups that were not formed by randomization. Thus, switchers and continuers could have differed in meaningful ways other than in the outcomes of switching. Dosing of patients was lower in switchers vs. continuers - though it is not clear whether this is due to patient factors or driven by the response to the assigned treatments. We conducted propensity score matching to help control for potential bias; however, this method accounts for a finite number of confounding factors measured at baseline and cannot fully adjust for all potential factors. In addition, the number of patients undergoing a switch from one medication or class of medications to another was small; therefore, data from all patients switching medications were combined into a single cohort. Conceivably, all switch combinations were not equally effective and/or well tolerated, and certain individual switches could have resulted in higher or lower acute-care service use compared with the pooled data. We also were not able to fully assess reasons for antipsychotic switches, which may be driven by diverse factors, including lack of medication efficacy, medication intolerability, and other patient-related factors [3,9,20].

In the present study, approximately one-third of patients who continued on their original regimens discontinued the study prematurely without switching. Although data from a sensitivity analysis that excluded discontinued patients were consistent with our core findings, it is difficult to delineate the potential switching patterns for these patients and the potential impacts of these changes on their subsequent psychiatric care. Costs and crisis events were prorated for this group to provide a 1-year estimate; however, in a usual-care treatment setting, some of these patients might have found a different, more effective or better-tolerated, antipsychotic agent. Consequently, these prorated costs may have overestimated the actual costs of switching. On the other hand, other patients who discontinued antipsychotic treatment prematurely may have remained without an effective therapy for some period of time, such that our prorated costs might have underestimated actual charges. Sensitivity analyses regarding patients who were randomized to the same therapy they had discontinued just prior to the trial were consistent with the reported results and directionally consistent with previous research [12]. However, sample size limited full assessment of the findings within patient groups based on prior therapy.

By design, clinical data were gathered only at specific assessment points throughout the year, limiting our ability to assess clinical changes at the time of each medication switch. In addition, the study had a 1-year time horizon, which may introduce other clinical and cost implications. For example, treatment-emergent weight gain and metabolic or endocrine changes may impact patients' resource utilization for several years after antipsychotic regimen changes [10,23,24]. Finally, the present analysis included only direct costs and did not account for lost productivity of patients and caregivers and other indirect societal costs.

## Conclusion

In conclusion, switching antipsychotic medications is common in the management of schizophrenia and is associated with crisis events, which are costly in both human and economic terms. Tailoring antipsychotic treatment to the individual needs of patients and monitoring the effectiveness of such therapy may help to optimize treatment responses and limit adverse consequences. Further research is warranted to identify reliable patient- and treatment-related predictors of antipsychotic switching.

## Competing interests

This study was funded by Eli Lilly and Company, Indianapolis, IN USA. The authors are full-time employees of Lilly and minor shareholders (stock/options).

## Authors' contributions

DEF participated in the study design, acquisition of data, analysis of data, and manuscript preparation. HA-S had oversight of the study design and participated in the manuscript preparation. AWN participated in the study design, acquisition of data, and analysis of data. BJK had oversight of the study design. All authors participated in the interpretation of the data and read and approved the final manuscript.

## Pre-publication history

The pre-publication history for this paper can be accessed here:


